# Cohort Profile: A European Multidisciplinary Network for the Fight against HIV Drug Resistance (EuResist Network)

**DOI:** 10.3390/tropicalmed8050243

**Published:** 2023-04-23

**Authors:** Barbara Rossetti, Francesca Incardona, Giulia Di Teodoro, Chiara Mommo, Francesco Saladini, Rolf Kaiser, Anders Sönnerborg, Thomas Lengauer, Maurizio Zazzi

**Affiliations:** 1 Infectious Diseases Department, Infectious Diseases Unit, USL SUDEST Toscana, Misericordia Hospital, 58100 Grosseto, Italy; 2 EuResist Network, 00152 Rome, Italy; 3 I-PRO, 00152 Rome, Italy; 4 Department of Computer Control and Management Engineering Antonio Ruberti, Sapienza University of Rome, 00185 Rome, Italy; 5 Department of Medical Biotechnologies, University of Siena, 53100 Siena, Italy; 6 Institute of Virology, University and University Hospital Cologne, University of Cologne, 50923 Cologne, Germany; 7 Department of Medicine Huddinge, Karolinska Institutet, Division of Infectious Diseases, 17177 Stockholm, Sweden; 8 Department of Laboratory Medicine, Karolinska Institutet, Division of Clinical Microbiology, 17177 Stockholm, Sweden

**Keywords:** HIV, HIV subtypes, antiretroviral therapy, drug resistance, treatment-response prediction system

## Abstract

The EuResist cohort was established in 2006 with the purpose of developing a clinical decision-support tool predicting the most effective antiretroviral therapy (ART) for persons living with HIV (PLWH), based on their clinical and virological data. Further to continuous extensive data collection from several European countries, the EuResist cohort later widened its activity to the more general area of antiretroviral treatment resistance with a focus on virus evolution. The EuResist cohort has retrospectively enrolled PLWH, both treatment-naïve and treatment-experienced, under clinical follow-up from 1998, in nine national cohorts across Europe and beyond, and this article is an overview of its achievement. A clinically oriented treatment-response prediction system was released and made available online in 2008. Clinical and virological data have been collected from more than one hundred thousand PLWH, allowing for a number of studies on the response to treatment, selection and spread of resistance-associated mutations and the circulation of viral subtypes. Drawing from its interdisciplinary vocation, EuResist will continue to investigate clinical response to antiretroviral treatment against HIV and monitor the development and circulation of HIV drug resistance in clinical settings, along with the development of novel drugs and the introduction of new treatment strategies. The support of artificial intelligence in these activities is essential.

## 1. Introduction 

Although a cure for HIV cannot be achieved with available antiretrovirals (ARVs), the last 25 years saw dramatic improvement in the prognosis and management of HIV infection, thanks to the use of multiple drugs from different antiretroviral classes [1,2,3]. Indeed, combined antiretroviral therapy (cART) currently allows for the achievement and maintenance of virological suppression in most people living with HIV (PLWH), halting disease progression and contributing to preventing HIV transmission [4,5].

As longer periods of follow-up data have become available, the clinician’s attention has shifted towards treatment tolerability and durability. Nevertheless, the impact of pre-existent drug-resistant mutations has remained a challenge in clinical practice, especially in PLWH exposed to suboptimal regimens in the first ART era [6]. In addition, drug-resistance may still variably emerge at virological failure under any ARV combination, due to suboptimal adherence to treatment, as well as low forgiveness, pharmacokinetic interactions and the variable genetic barrier of the virus. In turn, drug-resistant strains can be transmitted to newly infected people, reducing future treatment options [7]. 

In 2006, a multidisciplinary group of scientists created the EuResist consortium to fight ARV resistance and obtained a grant by the European Commission under the 6th Framework Programme. The project integrated three existing national cohorts from Italy, Germany and Sweden to form a large dataset of clinical and virological information from around 17,000 PLWH, thus giving rise to the EuResist Integrated DataBase (EIDB). By modeling these data via statistical learning, the EuResist project delivered a clinical decision-support tool for predicting the best ART for specific PLWH by leveraging curated real-world data. 

## 2. Cohort Description

The multicohort EuResist Network was established in 2008 as a follow-up of the EuResist project to manage the EIDB and the EuResist ART response prediction system. Currently, the EIDB is one of the world’s largest datasets suitable for training genotype-centered treatment-response models. The EIDB collects information about pseudonymized demographic and clinical characteristics of PLWH, including antiretroviral therapies, reasons for treatment change, AIDS defining events, viral co-infections, CD4 + T cells counts, viral load measurements, and HIV sequences.

**Figure 1:**
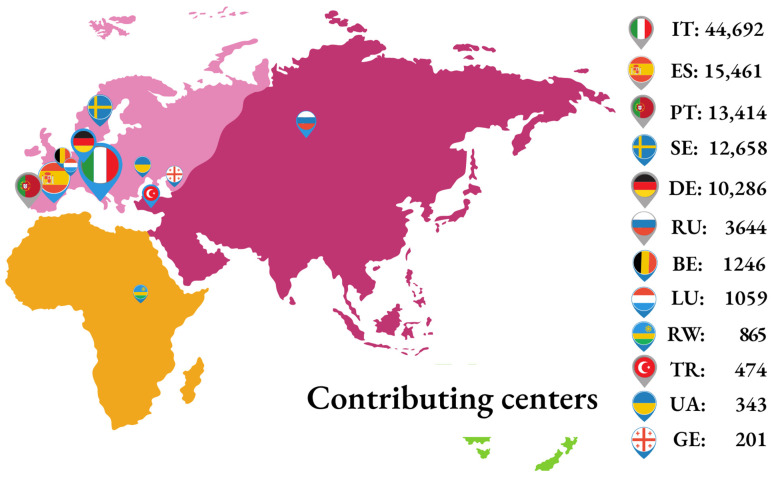
Number of patients contributed by the different participating centers (updated March 2022). Further to these, two clinical studies contributed their data, including the DUET study (Tibotec, 200 patients) and the Resist study (Boehringer Ingelheim, 1361 patients).

Originally, the EIDB was developed by integrating biomedical information from the three founding nationwide databases: ARCA (Italy), AREVIR (Germany), and InfCareHIV at Karolinska Institute, Stockholm (Sweden). Following the initial setup, other centers became partners of the EuResist Network and provided biomedical data to the EIDB. Most of these remain active contributors, together with the three founders; at time of writing, this includes: the Instituto de Higiene e Medicina Tropical (Portugal); the IrsiCaixa Foundation (Spain); the Laboratoire de Rétrovirologie of CRP-Santé (Luxembourg); the Gamaleya Institute of Virology (Russia); the Koacaeli University Medical Faculty (Turkey); and the CoRIS cohort (Spain). Additionally, data from the Rega Institute (Belgium); the National Referral Laboratory of Rwanda; the DUET study conducted by Tibotec; and the Resist study conducted by Boehringer Ingelheim, from the Infectious Diseases, AIDS and Clinical Immunology Research Centre of the State Institution Centre Public Health Ministry of Health of Ukraine and from the Infectious Diseases AIDS and Clinical Immunology Research Centre (IDACIRC) of Georgia were integrated in the past (Figure 1). 

**Figure 2:**
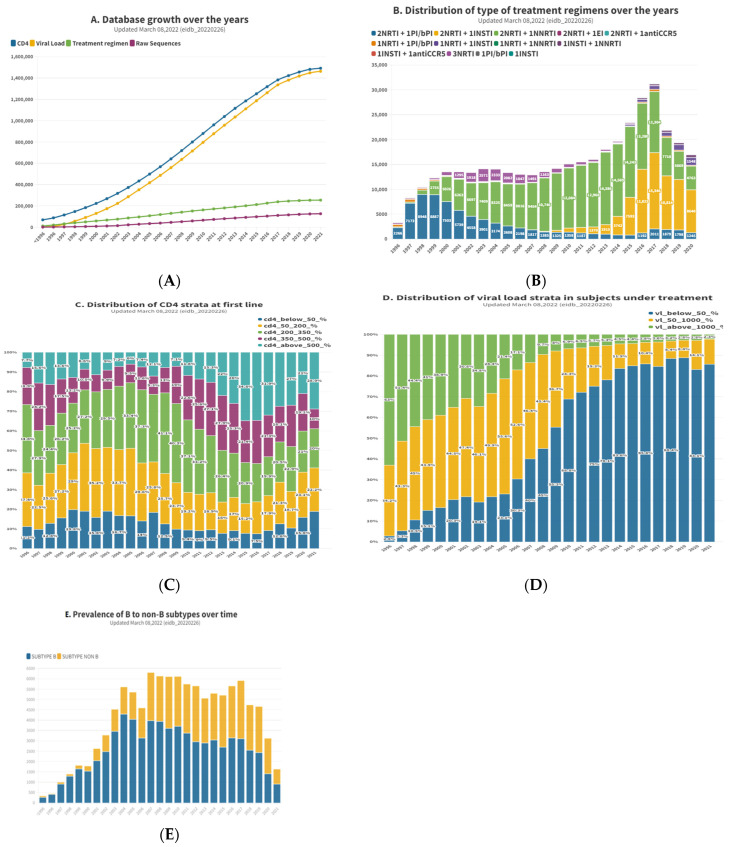
Main features of the enrolled PLWH at last available update (March 2022). ((**A**). Database growth over the years; (**B**). Distribution of type of treatment regimens over the years; (**C**). Distribution of CD4 strata at first line; (**D**). Distribution of viral load strata in subjects under treatment; (**E**). Prevalence of B to non-B subtypes over time).

**Figure 3:**
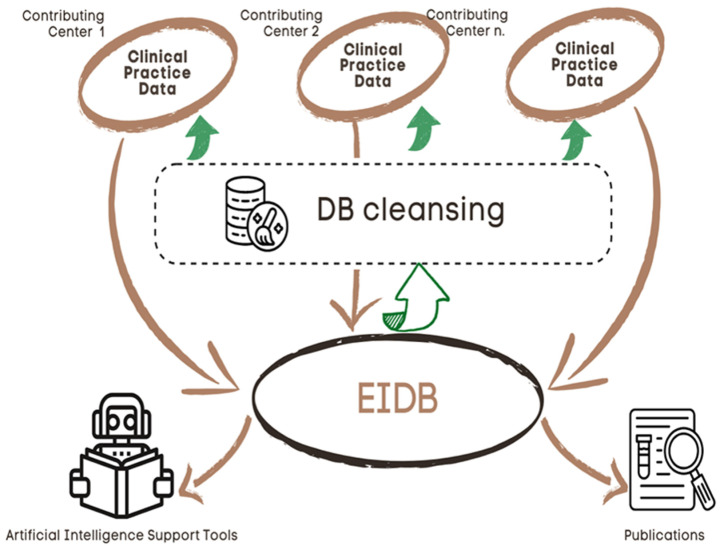
EuResist DB computation system.

The EIDB is updated twice a year with new data from the contributing centers by using import routines maintained centrally by the EuResist IT staff. Currently, it contains data from 105,903 PLWH, contributing 102,851 viral sequences, 248,249 treatment regimens, 1,451,753 viral load measurements and 1,486,295 CD4 cell counts [Figure 2A]. The main characteristics of the population are shown in Table 1, while Figure 2 illustrates the change over time for a few variables, representative of a complete series of graphs available at (https://www.euresist.org/eidb accessed on 31 March 2022). Data integrity and updating have been ensured for the last 15 years and onwards through an iterative process of data cleansing and periodical refresh from the contributing centers. The key uses of the EIDB have included the training of a machine-learning-based engine assisting treatment choices and providing curated datasets for specific studies, focusing on HIV variability, treatment, and drug resistance [Figure 3].

The objective of this manuscript is to offer an overview of the main contribution provided by the EuResist multicohort initiative in the fight against HIV drug resistance during the last 15 years, based on the efforts of many researchers from different European countries.

## 3. Data Availability Statement

The EuResist Network complies with the General Data Protection Regulation (GDPR) and all applicable laws, rules and regulations, with particular reference to ethical issues concerning the collection of data belonging to HIV patients and the relative manipulation and sharing procedures. All the data contributors to the EIDB certify that: the data collection was approved by the local ethics committees in agreement with local requirements and written informed consent was obtained from all patients before participation when necessary; the procedures are performed under the guidance and responsibility of the contributing centers; personal data are lawfully processed and may be contributed to the EIDB for scientific purposes on the basis of the data subject’s consent or another legal basis valid under the GDPR (art. 6 and art. 9).

This study is performed in accordance with the ethical principles of the Declaration of Helsinki and the Good Clinical Practice guidelines of the International Conference on Harmonization.

Data are securely stored in Italy. They contain sensitive human subject information and cannot be shared publicly because of privacy issues. Data are available under request to the EuResist Scientific Board, comprising all the data controllers. 

## 4. Patient and Public Involvement

The EuResist Network has received research grants from the European Commission and unrestricted research grants from the following pharmaceutical companies: Abbott, Janssen Tibotec, Gilead Sciences, Merck Sharp and Dohme, Pfizer, Theratechnologies, and ViiV Healthcare. Neither the companies nor the patients had any role in the design, recruitment, or conduct of the studies.

## 5. Findings to Date

### 5.1. Prediction of Response to Treatment

The emergence of antiretroviral drug resistance has long been a leading cause of treatment failure in PLWH. Particularly, before the advent of compact, high-genetic-barrier cART, the development of drug resistance was almost inevitable at some point in time. Thus, strategies for delaying the onset of resistance, preserving treatment options and avoiding cross-resistance were typically aided by algorithms interpreting HIV genotype. While these systems were mostly based on expert reviews of the literature, the EuResist consortium proposed a novel approach for predicting the in-vivo efficacy of antiretroviral drug regimens against a given virus isolate, based on statistical learning from a large amount of data correlating virus genotype and patient data on treatment response in clinical practice. 

Based on the analysis of the EIDB data, different predictive models have been developed over the years. In 2008, the first machine-learning engine was reported, which combined three models as follows: (a) generative-discriminative method, (b) regression with derived evolutionary features and (c) regression with a mixture of effects. The engine demonstrated an increase in the accuracy of prediction when taking into account clinical information and demographic factors in addition to genotypic information [8]. The system underwent a formal assessment in the pilot EVE (EuResist *vs* human Experts) study in which 25 complete patient histories were provided to a panel of 10 global top experts in HIV drug resistance, who had to indicate whether the next ART regimen used would be effective or not. The EuResist system outcompeted nine of the experts in accuracy and equaled the most proficient one, demonstrating the strength and feasibility of the approach in a real-world simulation [9]. 

In 2010, Bogojeska et al. [10] developed a method for predicting the outcome of a therapy, circumventing the problem of data sparsity, with a multi-task hierarchical Bayes setting where the tasks were the different drugs that belong to the target therapy. In 2011, Saigo et al. [11] developed an approach that takes into account the treatment history of each patient. This idea was further developed by Bogojeska et al. [12] based on a similarity measure between longitudinal sequences of therapies administered to a patient, which amounts to the score of an alignment of two therapy sequences that is calculated in a similar fashion as is customary with genome sequences. The resulting score between the input and a training sample provides a weight with which that sample enters a regularized logistic regression, thus giving training samples with therapy sequences more similar to that of the input higher impact on the final result. The linearity and sparsity of the model also allows the interpretability of the predictions, which is important in the medical field given the reluctance to accept predictions from black-box systems. In addition, Bogojeska et al. [13] presented prediction methods that are sensitive to the uneven representation of different combination drug therapies in the database. In the same years, Weisser et al. [14] showed that the replicative capacity of the virus can be predicted using a support vector machine and that its introduction in a linear regression model using information on the drug combination and the genotypic sensitivity score only slightly increases the ability to predict the response to treatment. Sangeda et al. [15] investigated the predictive role of a viral fitness estimate in a representative dataset of PLWH treated with indinavir and demonstrated that the fitness landscapes have similar predictive power for treatment response as standard rules-based algorithms, additionally allowing us to predict genetic evolution under indinavir selective pressure. In 2014, El-Hay et al. [16] introduced a conditional probabilistic model for learning the association between the observed variables of PLWH and future response to treatment that is scalable and provides a clear expression of the interrelationship between the components of the system. Prosperi et al. [17] developed and implemented a model for predicting the duration of effectiveness of an ART therapy using multiple input domains (demographics, clinical, laboratory and virus genetics). De Luca et al. [18] explored a model with a new darunavir-weighted mutation score, outperforming the reference genotype interpretation systems in predicting virological response to darunavir on both B and non-B subtypes. In 2017, Libin et al. [19] developed the PhyloGeoTool, a visual method for exploring the epidemic spread of HIV variants using viral sequences in the EuResist database. The method analyzes large phylogenetic trees and the characteristics of strains and clades, together with their geographical context, offering the possibility to add new strains without having to reconstruct the entire phylogeny. The EIDB was used in 2017 by Wu et al. [20] to test deep learning models regularized in such a way that they assume a form of interpretability termed human simulability, i.e., deep learning models whose class probability predictions on therapy outcome have a high accuracy and are modeled by decision trees with few nodes so that the decision of the model can be interpreted by a human following only few decision steps. Parbhoo et al. [21,22] mixed kernel- and model-based techniques to capture both the similarity of clusters of patients via the kernel method and to predict accurate patterns of the patients’ response to treatment for the patients outside these clusters. 

It must be noted that, despite considerable efforts to improve the algorithms for prediction of response to ART, the increase in accuracy with respect to standard systems based only on the interpretation of HIV genotype has been limited. Factors impeding higher benefits by applying advanced statistics have not been identified but likely include the sparse representation of data in the training datasets and the complete lack of data on one major determinant of response to treatment, namely adherence to therapy. Moreover, the gain in accuracy comes at the cost of additional data to be input into the system—something that most doctors are reluctant to do. Additional bottlenecks for the transition from simple to improved yet more-complex methods include the lower degree of human interpretability of the latter and the globally decreased impact of drug resistance in modern ART [23]. As a matter of fact, the pivotal role of the EuResist engine demonstrated the feasibility and added value of machine learning in assisting HIV treatment choices. It did not result in further developments, and the online engine was later discontinued. For HIV genotype interpretation, clinicians still rely on simpler services available on the web, particularly the Stanford HIVdb system, which provides a five-level ranking of activity for each HIV drug once the user has submitted an HIV sequence or a list of mutations. While the HIVdb system is hand-crafted by experts and does not resort to data analytics, it has gained long-term popularity and remains the leading system in the clinic. Of note, the Stanford website (https://hivdb.stanford.edu/ accessed on 10 April 2023) provides an impressive number of options for querying a curated HIV sequence database derived from published HIV sequences, with novel features and services released on a regular basis.

### 5.2. Using Real World Data to Assess the Impact of Drug Resistance on Clinical Outcome 

Thanks to the continuous and longitudinal accumulation of viral sequences and patient data, the EIDB has offered a tremendous opportunity to monitor the selection and spread of transmitted and acquired resistance-associated mutations (RAMs), the circulation of different viral subtypes, and their impact on treatment outcome, most often as measured by changes in viral load. In more recent years, non-B subtypes have become endemic in the European population, likely due to human migration from non-B areas. This may have had an impact on the global picture of drug resistance since most non-B areas have been characterized by a different coverage of ART regimens and consequently different patterns of emergent drug resistance. This effect has been later diluted and will continue to be diluted by the harmonization of ART regimens, particularly those based on high-genetic-barrier regimens, significantly limiting drug resistance and failure.

A key issue in HIV drug resistance is the prevalence and incidence of multidrug resistance, resulting in an extensive loss of treatment options and the inability to control virus replication and halt disease progression. Two EuResist studies have shown a declining trend of resistance to the four main classes of ARVs, including protease inhibitors (PI), nucleoside (NRTI) and non-nucleoside (NNRTI) reverse transcriptase inhibitors, and integrase strand transfer inhibitors (INSTI). The first study analyzed viral sequences collected from 3414 individuals with at least 1 genotype test result available between 2008 and 2019 and found that high-level resistance to at least 1 drug in each of the 4 main ARV classes declined from 5.6% in 2008 to 2.4% in 2018, with only 2.5% of the patients accumulating four-class resistance over time [24]. Notably, this analysis only considered PLWH who underwent resistance testing for all of the four drug classes. Since analysis of INSTI susceptibility before starting ART has not become standard of care, this dataset mostly included patients failing an INSTI-based ART, ignoring all those PLWH not experiencing this event and likely to harbor an INSTI-susceptible virus. To overcome this selection bias and deliver a broader overview of multidrug resistance, the second study analyzed the incidence and prevalence of three-class (PI, NRTI and NNRTI) resistance between 1996 and 2019 in 39,956 PLWH, and four-class resistance (PI, NRTI, NNRTI and INSTI) between 2008 and 2019 (when INSTI were available) among 16,019 cases. Dissimilarly to the previous study, this analysis included PLWH receiving ART even in the absence of integrase genotypic data, assuming that resistance to INSTI was not present without exposure to INSTI but emerged at therapy failure under particular conditions (i.e., use of the first-generation INSTIs raltegravir and elvitegravir). With these assumptions, three-class resistance had developed in 6.9% of individuals on treatment and since the introduction of INSTI in 2008, less than 2% of PLWH had developed four-class drug resistance [25].

The studies conducted by Lapokov et al. [26] and Neshumaev et al. [27] explored the prevalence of viral subtypes and INSTI RAMs in isolates circulating in Russia and surrounding countries. The phylogenetic analyses based on the integrase sequences revealed that sub-subtype A6 was the most prevalent variant, presumably following introduction of a single viral lineage into the Krasnoyarsk region, which occurred around September 1996 (May 1994–May 1999) and then spread to the neighboring districts. Because of the low prevalence of INSTI RAMs, the study also suggests a very low risk of initiating INSTI-based therapy in patients with pre-existing reduced susceptibility to INSTI in Russia and other countries belonging to the former Soviet Union. 

A recent study conducted by van de Klundert et al. [28] showed, in contrast to earlier studies, no clear clusters related to the route of transmission, indicating that, within Eastern Europe and Russia, the exchange of viruses among the different risk groups may occur more often than earlier reported.

Over the years, the EIDB was used to explore the clinical impact of specific RAMs. For example, Theys et al. [29] observed that the emergence of K65R in the reverse transcriptase coding region was significantly favored in HIV-1 subtype C in PLWH being treated with tenofovir, suggesting subtype-specific pathways that facilitate the emergence of an otherwise-rare RAM. More recently, Kuznetsova et al. [30] identified 11 individuals in the EuResist database harboring the reverse transcriptase natural polymorphism E138A who started a first-line ART including rilpivirine. Even though the variant E138A moderately decreases susceptibility to RPV in vitro and is included in the list of rilpivirine RAMs in several algorithms, treatment with RPV-based ART was fully effective in all 11 patients, supporting the effectiveness of RPV in first-line therapy in countries such as those belonging to the Russian Federation, where the natural polymorphism E138A is present in 4–8% of circulating strains.

Inspired by the updates of the international HIV treatment guidelines, the EuResist Network explored the real-world effectiveness of different treatment strategies. Understanding and predicting treatment efficacy and duration of effectiveness, i.e., how long an antiretroviral therapy can be sustained without changes, even in the presence of pre-existing RAMs, has become an important challenge in the context of addressing HIV treatment optimization. For example, the analysis of the EIDB data suggested that the efficacy of the innovative dual-drug strategy combining the NNRTI etravirine with the ritonavir-boosted PI darunavir was comparable to that of alternative regimens where etravirine was coupled with an alternative boosted PI [31]. In 2019, De Luca et al. [32] aimed to survey maraviroc use and assess the effectiveness and durability of maraviroc-containing ART in routine practice across Europe. The study, the largest of this kind, included 1381 PLWH who started maraviroc across 8 European countries. In this overall highly treatment-experienced population, with a small but appreciable subset of patients that received maraviroc outside of standard treatment guidelines, maraviroc was safe and reasonably effective, with relatively low rates of discontinuation over 48 weeks and only 2 cases of serum transaminase elevations reported as reasons for discontinuation. Still in 2019, Borghetti et al. [33] demonstrated that the genotypic susceptibility to the NRTI backbone is desirable when prescribing an INSTI-based ART. Indeed, among 1095 PLWH, pre-treatment NRTI resistance increased the risk of virological failure with the first-generation INSTI raltegravir but not with the second-generation INSTI dolutegravir-based regimens. The M184V/I mutation independently predicted the virological failure of raltegravir- but not of dolutegravir-based therapy when compared with a fully active backbone, particularly when associated with other non-thymidine analogue mutations. Higher-zenith HIV-RNA and lower nadir CD4 counts also independently predicted virological failure. 

More recently, two studies explored the effectiveness and discontinuation rate of INSTI, a key component of most current ART regimens. Among 2976 INSTI treatments started by naïve PLWH, elvitegravir- or dolutegravir-based first-line antiretroviral treatment was highly effective in routine practice across Europe, even in the presence of transmitted drug resistance for the accompanying NRTIs. However, the rate of INSTI discontinuation was much higher than the rate of virological failure [34]. Interestingly, among almost 14,000 INSTI-based treatments started by pre-treated, viremic, or non-viremic individuals, including both INSTI-experienced and INSTI-naïve subjects, a very low rate of INSTI resistance mutations among INSTI-naïve individuals—less than 1%—was confirmed. The study demonstrated the overall high virological efficacy of the INSTI-based ART regimens started between 2012 and 2019, with a higher risk of virological failure for raltegravir-based regimens and in cases with worse clinical indicators at baseline. The risk of INSTI discontinuation was higher among viremic individuals and predicted by the use of first-generation INSTI, reflecting the time-dependent availability of second-generation INSTI and some single-tablet regimens [35].

Another study by Miranda et al. [36] investigated the clinical and socio-demographic information from 89,851 HIV-1 infected patients (1981–2019), aiming to characterize the features associated with late HIV diagnosis. Among the study population, 28,889 patients (50.4%) were late presenters (defined as CD4 <350 cell/mmc). Older patients (>56 years), heterosexuals, those originating from Africa and patients presenting with log VL > 4.1 had a higher probability of being late presenters. 

### 5.3. Studies in Low/Middle Income Countries

The EuResist Network has been collaborating with other non-European cohorts and global initiatives to investigate HIV-resistance-related issues, particularly in low-income countries. From 2010 to 2013 it participated in the ENEAA project (EDCTP JP.2009.10800.002), coordinated by Karolinska Institut and involving the Addis Ababa University, Ethiopia; the Muhumbili hospital, Dar-es-Salaam, Tanzania; and the World Friends Hospital, Nairobi, Kenya, integrating training activities and IT infrastructures to improve capacities in the eastern African area. The TenoRes collaboration included data from adult HIV treatment cohorts and clinical trials in Europe, Latin and North America, sub-Saharan Africa, and Asia. Among 1926 PLWH enrolled after virological failure with first-line tenofovir-containing ART, HIV drug resistance was detected in an alarmingly high fraction of cases, strengthening the need for the surveillance of transmitted drug resistance [37]. Another study based on four ART centers in Ethiopia, Ghana, and Uganda, focused on second-line ART failure between 2005 and 2017. In a total of 2191 subjects included in the study, the effectiveness of the second-line ART regimens was good but was challenged by interactions with TB therapy [38].

## 6. Collaboration

The EIDB can be accessed upon formal request by scientists aiming at investigating HIV treatment and drug-resistance-related topics. The EuResist Management Board processes requests in a reasonable time and asks the individual cohorts for permission to access the data for the specific study, which has to be well-defined and scientifically sound. EuResist encourages other clinicians and scientists, also from non-European countries, to collaborate for the advancement of knowledge on HIV drug resistance and treatment, and it is open for scientific studies. 

## 7. Further Details 

EuResist Network is a non-profit European Economic Interest Grouping (EEIG) composed by:

- Karolinska Institutet (Stockholm, Sweden) [39];
- Max Planck Gesellschaft (Germany);
- University of Siena (Italy);
- InformaPRO s.r.l. (Rome, Italy);
- Cologne University (Germany).

In addition to the already mentioned EuResist project (Grant n. IST-2004-027173, 6^th^ Framework Programme (FP)), the EuResist Network participated in and guided several research projects funded by the European Commission, among which we can mention: CHAIN-Collaborative HIV and Anti-HIV drug resistance Network, a large scale integrated project (grant No Health-2007-223131, VII FP); CARE-Common Action against HIV-TB HCV across the Regions of Europe, (grant No 825673, Horizon 2020); and EuCARE-European Cohorts of Patients and Schools to Advance Response to Epidemics (grant No 101046016, Horizon Europe), where the focus moves to SARS-CoV-2 ad its variants.

EuResist also runs dissemination and training activities. Further to the cited ENEAA EDCTP project, it offers training tools on its website; it participates in the annual ARCA Mentor School event, and has organized a EuResist Mentor School in Cologne in 2018 with the objective to bridge the gap between data science and medical/biological sciences.

Since 2007, EuResist has contributed to the annual Arevir Meeting organized by the University of Cologne and Genafor, holding a EuResist session.

## 8. Strengths and Limitations 

The main strength of the EuResist Network is the inclusion of all PLWH followed at each of the participating centers, ensuring the full representativeness of the real-life setting, including individuals typically not enrolled in randomized trials due to, e.g., the presence of baseline drug resistance or past virological failures. The sample size of the cohort is also notable, with a total of 105,903 PLWH as of March 2022. Likewise, the geographic distribution of the participating centers is Europe-wide, with additional cases from Turkey, Russia, Ukraine, Georgia, and Rwanda. The observational, retrospective, and prospective multicohort design provides an extraordinarily large time span, with data collection starting as early as the late 1980s, translating into a unique opportunity to follow virus evolution and investigate very different treatment strategies across all antiretroviral treatment eras.

The main limitation of the EIDB is that most contributing cohorts collect only routine clinical and viro-immunological data. Some data can be missing in the individual databases, making the overall data collection not uniform across areas and resulting in the exclusion of large datasets when specific variables are required. The lack of information on tolerability issues, adherence to ART, clinical non-AIDS-related events and concomitant medications make the cohort not suitable for many kinds of investigations. Lastly, although a semiannual update is planned, delays between local data collection and the availability of an updated centralized database often occur, postponing the possibility to study the most recent treatments, which are typically of maximum interest.

## 9. Conclusions

The EuResist cohort was established with the purpose of developing a clinical decision-support tool predicting the most effective antiretroviral therapy for PLWH based on their clinical and virological data. Later, the cohort widened its activity to the more general area of antiretroviral treatment with a focus on virus evolution and drug resistance. Following its interdisciplinary vocation, EuResist will continue to investigate clinical response to antiretroviral treatment against HIV and monitor the development and circulation of HIV drug resistance in the clinical setting along with the development of novel drugs and the introduction of new treatment strategies. The support of artificial intelligence in these activities is essential. 

## Figures and Tables

**Table 1:** Main features of the enrolled PLWH at last available update (March 2022).

Variable	Overall (*n* = 105,903)
Male gender, n (%)	71,041 (67.08%)
Female gender, n (%)	26,953 (25.45%)
Unknown gender, n (%)	7909 (7.47%)
Age (years), median (IQR)	53 (44–61)
Median calendar year at first genotype (IQR)	39 (32–47)
Nadir CD4+ (cells/mmc), median (IQR)	476 (304–678)
Zenith HIV-RNA (log_10_ copies/mL), median (IQR)	4.85 (3.91–5.42)
PR/RT sequences, n (%)	87,339 (69.26%)
IN sequences, n (%)	15,512 (12.30%)
Viral subtype B, n (%)	69,463 (57.34%)
3-drugs ARV regimens, n (%)	121,729 (75.46%)
<3-drugs ARV regimens, n (%)	43,949 (17.30%)
>3-drugs ARV regimens, n (%)	18,391 (7.24%)
Treatment-naïve at EIDB entry, n (%)	13,827 (13.06%)
Treatment-experienced at EIDB entry, n (%)	64,687 (61.08%)
Unknown treatment status at EIDB entry, n (%)	27,389 (25.86%)

## Data Availability

Data are available under request to the EuResist Scientific Board, comprising all the data controllers.
